# Autophagy-based survival prognosis in human colorectal carcinoma

**DOI:** 10.18632/oncotarget.3054

**Published:** 2015-01-20

**Authors:** Maopeng Yang, Hong Zhao, Li Guo, Qingyuan Zhang, Ling Zhao, Shuping Bai, Minghui Zhang, Sanqi Xu, Fujing Wang, Xiaohong Wang, Bin Zhao

**Affiliations:** ^1^ Department of Medical Oncology, The Third Affiliated Hospital of Harbin Medical University, Heilongjiang, China; ^2^ Department of General Surgery, The Second Affiliated Hospital of Harbin Medical University, Heilongjiang, China; ^3^ College of Basic Medicine, Harbin Medical University-Daqing, Heilongjiang, China

**Keywords:** Colorectal carcinoma, autophagy, Beclin 1, LC 3B, Bcl-xL

## Abstract

The role of autophagy in cancers is controversial. Here we aim to determine the prognostic significance of autophagy in colorectal carcinoma patients, thereby allowing more rational development of therapeutic strategies. Through transmission electron microscopy, our data first demonstrated high frequency of defective mitochondria was strongly associated with poor overall survival in colorectal carcinoma. Next immunohistochemical study showed the expressions of Beclin 1, LC3B and Bcl-xL in both the center of tumor and adjacent noncancerous mucosal region were also correlated with overall survivals. We developed an autophagy signature for prognosis based on these three major autophagic proteins, further analysis suggested it was an independent prognostic biomarker and had its value even within single clinical stage. Combined TNM stage and this signature could significantly improve the accuracy of survival prognosis. To validate these immunohistochemical results, an internal testing cohort and an independent population were also included. Our findings suggest that autophagy plays an important role in the clinical cancer progression. Therefore autophagic proteins may be valuable prognostic biomarkers in the therapy of colorectal carcinoma and possibly other types of cancers.

## INTRODUCTION

Colorectal carcinoma (CRC) is the fourth leading cause of cancer related death in the world [1]. It is more common in developed countries. However, the incidence and mortality in some developing countries, such as China, have continued to increase because their transition towards the so-called western lifestyle such as the consumption of high-fat diets and physical inactivity, and relatively poorer health-care resources. Although great progress has been achieved in the past decade, CRC patient survival is still poor [2]. Accordingly it is of substantial value to understand the pathogenic mechanisms and to figure out new prognostic biomarkers not only because it could improve poor prognosis but also provide novel potential targets for therapy.

Autophagy was discovered by transmission electron microscopy(TEM) over 50 years ago [3], it is a conserved pathway which degrades and recycles organelles (such as mitochondria) and proteins to generate amino acids, ATP, nucleotides, fatty acids and sugars to support cell survival [4]. Autophagy can exert multifactorial influence on tumorigenesis, tumor progression and cancer therapeutics [5]. Dysfunctional mitochondria in tumor was first observed by Warburg to explain that tumor cells undergo increased aerobic glycolysis (“Warburg effect”) compared to normal cells [6]. It remains enigma for decades why cancer cells would use such an inefficient process to meet their energy demands. Not until recently it is demonstrated that reactive oxygen species produced by tumor cells are transferred to the cellular microenvironment including supporting host cells such as adipocytes, endothelia, fibroblasts, smooth muscle cells and immune cells [7]. Then it can start the oxidative stress responses like autophagy in these cells, which lead to the production of high energy metabolites for the anabolic cancer cells to live and proliferation [7, 8]. This “parasitic cancer metabolism” model suggests that during the development of tumor, a large amount of dysfunctional mitochondria would occur for the survival of cancer cells.

Besides morphological visualization, now there are several proteins are commonly used as biomarkers of autophagosome formation [9]. As the first discovered autophagy effector in mammalian, Beclin 1 is has been demonstrated to be deleted or decreased mono-allelically in human breast, prostate and ovarian cancers [10–12]. Furthermore, several clinical researches associated aggressive tumor phenotypes and poor prognosis with aberrant expression of Beclin 1 [10, 13, 14]. In mice, deficiency or inactivation Beclin 1 can lead to a high incidence of tumors in liver, lung and lymphomas spontaneously [15, 16]. The important effects of Beclin 1 in the induction of autophagy may be attributed to its interaction with Bcl-xL [17]. As an anti-apoptotic protein in Bcl-2 family, B-cell lymphoma-extra-large (Bcl-xL) was first recognized as regulator in cell death and later had been characterized in controlling Beclin 1-mediated autophagy [18]. It has been reported low expression of Beclin 1 was associated with poor survival in Bcl-xL overexpressed ovarian cancer [19] and hepatocellular carcinoma [20]. Microtubule associated protein 1 light chain 3 (LC3) is a homolog protein of yeast Atg8 in mammalian [17]. During autophagic process, phosphatidylethanolamine is conjugated to LC3I (cytosolic form of LC3) to assemble LC3-II, which will then be recruited to autophagosomal membranes. Autophagosomes can fuse with lysosomes to create autolysosomes, and LC3-II in the autolysosomal lumen will be degraded [21]. As a result, the lysosomal turnover of LC3-II can directly reflect autophagic activity in cells. In particular, LC3 detection by immunofluorescence or immunoblotting are usually treated as a reliable method in monitoring autophagic process.

In this study, we examined the ultra-structural details of mitophagy by transmission electron microscopy, evaluated the expression level of Beclin 1, LC3B and Bcl-xL in both colorectal tumor and adjacent noncancerous mucosal tissues. We further developed an autophagy-protein-based classifier for survival prognosis and compared its efficacy to other clinical-pathological risk factors. The main purpose of this study was to explore the clinical significance of autophagy in CRC development and progression, and identify valuable prognostic biomarker, thereby allowing more rational development of therapeutic strategies against cancer.

## RESULTS

### Frequency of defective mitochondria and overall survival

**Figure 1:**
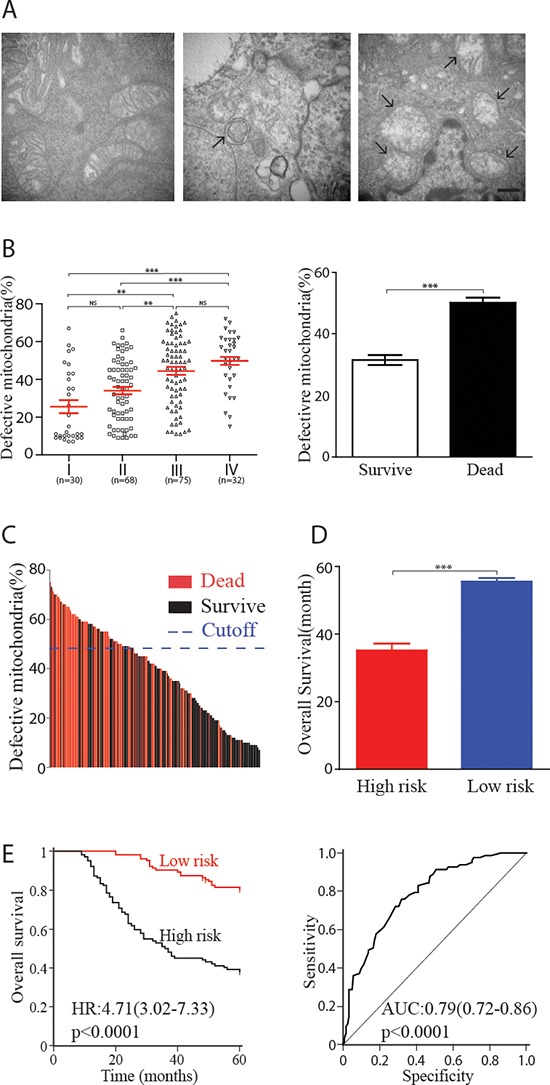
Defective mitochondria and clinical outcomes in human colorectal carcinoma **(A)** Examples of healthy mitochondria (left panel), autophagosome (middle panel) and defective mitochondria (right panel) observed in colorectal carcinoma. Scale bar = 500 nm. **(B)** Correlation between defective mitochondria percentage and clinical grading (left panel, each point represent one patient) or survival status (right panel). **(C)** 205 patients were classified into high-risk and low-risk subgroup based on the proportion of defective mitochondria, cutoff value was set at 50%. Each line represented one patient. **(D)** High-risk subjects showed worse survivals compare with low-risk patients (36.1 ± 2.3m vs.54.1 ± 1.0m). **(E)** Kaplan-Meier analysis of 5-year overall survival of high-risk and low-risk subject. ROC revealed the prognostic accuracy of 5-year survival was 0.79. HR, hazard ratio; AUC, area under the curve; ROC, receiver operating characteristics. **, *p* < 0.01; ***, *p* < 0.001; NS, *p* > 0.05

We first examined the morphology of mitochondria, one of the major substrates of basal autophagy [9], from 205 primary colorectal carcinoma tissues (Figure 1A). Defective mitochondria were observed in all tissues. The defective mitochondria were characterized by autophagosome, regular rupture in the outside part of membrane, increased size, and a clear matrix because of the cristolysis but without isolation membrane-like structures. Along with the clinical progress of cancer, the frequency of defective mitochondria elevated gradually. Moreover, tumors from dead subjects had higher proportion of defective mitochondria than survival patients (Figure 1B). Here, we classified all 205 patients into high-risk subgroup (defective mitochondria account > 50% of the total mitochondria) and low-risk subgroup (<50%) (Figure 1C). The distribution of the clinicopathological characteristics from these two subgroups was showed as Table 1. As expected, low-risk patients had better survival than high-risk subjects (Figure 1D). Kaplan-Meier survival curves analysis demonstrated that 5-year overall survival was 34% for the high-risk subgroup, and 75% for the low-risk subgroup (HR, 4.72, 95% CI, 3.04–7.32; *p* < 0.0001). Moreover, receiver operating characteristics (ROC) analysis suggested the prognostic accuracy of 5-year overall survival was 0.79 (95% CI, 0.73–0.85) (Figure 1E).

**Table 1:** Clinical characteristics of human CRC patients according to high- or low-risk TEM score

	patients with low risk (*n* = 126)	patients with high risk (*n* = 79)	*p* value
**Age, years**	58. 7(range, 34–82)	57. 6(range, 31–80)	0.24*
**Sex, male**	70(56%)	36(46%)	0.09
**Pathological type**			0.22
colon cancer	58(46%)	35(44%)	
rectal cancer	68(54%)	44(56%)	
**Family history of cancer**			0.27
yes	26(21%)	19(24%)	
no	100(79%)	60(76%)	
**T stage**			**0.01**
T1	5(4%)	0(0%)	
T2	25(20%)	9(11%)	
T3	67(53%)	47(59%)	
T4	29(23%)	23(30%)	
**N stage**			**< 0.0001**
NO	78(62%)	20(25%)	
N1	12(10%)	15(19%)	
N2	27(21%)	23(29%)	
N3	4(3%)	12(15%)	
Nx	5(4%)	9(11%)	
**TNM stage**			**< 0.0001**
I	25(20%)	5(6%)	
II	53(42%)	15(19%)	
III	37(29%)	38(48%)	
IV	11(9%)	21(27%)	
**CEA (ng/ml)**	14.98 ± 3.62	34.94 ± 5.94	**0.001**
**CA19–9 (U/ml)**	48.76 ± 8.35	127.38 + 18.50	**< 0.0001**
**Histology differentiation**			**< 0.0001**
well	88(70%)	11(14%)	
poorly	38(30%)	68(86%)	
**overall survival (5 year)**			**< 0.0001#**
alive	95(75%)	27(34%)	
dead	31(25%)	52(66%)	

Data are shown as n (%) or mean ± SE. *p* values are calculated by χ^2^ test or Fisher's exact test, unless otherwise stated.

* student's *t* test;

# Log-rank test;

TNM, tumor node metastasis; CEA, carcinoembryonic antigen; CA19–9, carbohydrate antigen 19–9.

### Expression of Beclin 1, LC3B and Bcl-xL and overall survival

Next we studied the expression of Beclin 1, LC3B and Bcl-xL in both the center of tumor (CT) area and noncancerous mucosal (NM) region. A total of 526 primary CRC subjects were enrolled. The mean age was 59 years (range 28–92 years), 261(50%) were males and 211 (40%) died during the follow-up period. The above 205 patients were assigned to the training set, additional 160 participants from the same hospital were included in the internal testing set, and 161 subjects from another hospital were treated as independent validation set.

**Figure 2:**
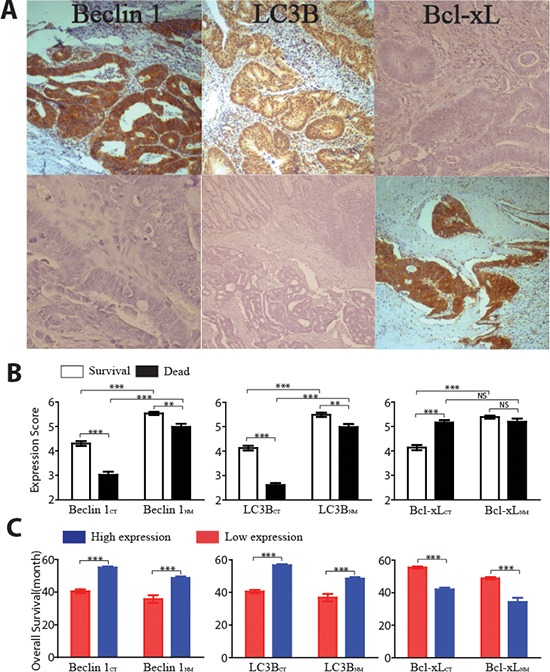
Overall survivals and expression of three autophagic proteins in both the center of tumor (CT) area and noncancerous mucosal (NM) region **(A)** Representative examples of Beclin 1, LC3B and Bcl-xL immunostaining in CT (lower panel) and NM (upper panel) (magnification, X200). **(B)** Comparison of the mean (±SE) of autophagic protein expression scores in CT and NM from patients who were dead (black bars) or patients who were survive (white bars). **(C)** Overall survival time for patients with high atuophagic protein expressions (blue bars) or low protein expressions (red bars) in CT or NM region. **, *p* < 0.01; ***, *p* < 0.001; NS, *p* > 0.05

Beclin 1 and LC3B (Figure 2A) were moderately expressed in CT area, but expressed robustly in NM region. Conversely, Bcl-xL showed the reversed expression patterns. As expected, the correlations among these three proteins were robust in the NM region (Beclin 1 vs. LC3B, *r* = 0.86, *p* < 0.001; Beclin 1 vs. Bcl-xL, *r* = –0.69, *p* < 0.001; Bcl-xL vs. LC3B, *r* = –0.65, *p* < 0.001). However, these correlations in CT weakened (Beclin 1 vs. LC3B, *r* = 0.41, *p* < 0.01; Beclin 1 vs. Bcl-xL, *r* = –0.32, *p* < 0.01; Bcl-xL vs. LC3B, *r* = –0.31, *p* < 0.01).

Tumor samples from subject who died had lower Beclin 1 and LC3B expressions within both CT and NM region compare with survival patients (Figure 2B). Interestingly, the Bcl-xL expression only showed statistical difference in CT region. To further assess OS, ROC curve analysis was conducted to figure out the optimum cutoff values [22] for Beclin 1_CT_ (3.79), Beclin 1_NM_ (4.52), LC3B_CT_ (4.34), LC3B_NM_ (4.58), Bcl-xL_CT_ (4.75) and Bcl-xL_NM_ (2.84) in the training set. These cutoff scores could segregate the enrolled 526 participants into high-expression and low-expression protein subgroups. The distribution of the clinicopathological characteristics from different subgroups was showed as Table 2–7. As shown in Figure 2C, higher levels of Beclin 1, LC3B and lower expression of Bcl-xL in either CT or NM regions were associated with better OS.

**Table 2:** Clinical characteristics of human CRC patients according to Beclin 1 expression in CT of the training, testing and independent sets

	training set			testing set			independent set		
	patients with low expression (*n* = 105)	patients with high expression (*n* = 100)	*p* value	patients with low expression (*n* = 76)	patients with high expression (*n* = 84)	*p* value	patients with low expression (*n* = 84)	patients with high expression (*n* = 77)	*p* value
**Age, years**	58.7(31–80)	57.9(34–82)	0.29	56.5(28–84)	58.5(31–83)	0.15	60.6(35–92)	62.6(35–84)	0.14
**Sex, male**	50(48%)	56(56%)	0.12	40(53%)	42(50%)	0.37	39(46%)	34(44%)	0.39
**Pathological type**			0.25			0.31			0.32
colon cancer	50(48%)	43(43%)		42(55%)	49(58%)		53(63%)	54(70%)	
rectal cancer	55(52%)	57(57%)		34(45%)	35(42%)		31(37%)	23(30%)	
**Family history of cancer**			0.16			0.4			0.12
yes	26(25%)	19(19%)		23(30%)	27(32%)		29(35%)	20(26%)	
no	79(75%)	81(81%)		53(70%)	57(68%)		55(65%)	57(74%)	
**T stage**			**0.0001**			**< 0.0001**			**< 0.0001**
T1	0(0%)	5(5%)		0(0%)	11(13%)		1(1%)	10(13%)	
T2	11(10%)	23(23%)		9(12%)	19(23%)		7(9%)	19(25%)	
T3	60(57%)	54(54%)		35(46%)	35(41%)		38(45%)	36(47%)	
T4	34(33%)	18(18%)		32(42%)	19(23%)		38(45%)	12(15%)	
**N stage**			**0.003**			**0.004**			**0.002**
N0	40(39%)	58(58%)		29(38%)	46(55%)		33(39%)	42(55%)	
N1	15(14%)	12(12%)		12(16%)	14(17%)		11(13%)	16(21%)	
N2	26(25%)	24(24%)		14(18%)	13(15%)		12(14%)	16(21%)	
N3	13(12%)	3(3%)		15(20%)	6(7%)		19(23%)	2(2%)	
Nx	11(10%)	3(3%)		6(8%)	5(6%)		9(11%)	1(1%)	
**TNM stage**			**< 0.0001**			**0.006**			**0.0005**
I	6(6%)	24(24%)		4(5%)	19(23%)		4(5%)	19(25%)	
II	34(32%)	34(34%)		25(33%)	27(32%)		29(35%)	23(30%)	
III	41(39%)	34(34%)		34(45%)	26(31%)		33(39%)	28(36%)	
IV	24(23%)	8(8%)		13(17%)	12(14%)		18(21%)	7(9%)	
**CEA (ng/ml)**	33.68 ± 5.79	11.11 ± 2.28	**0.0002**	34.28 ± 5.90	13.38 ± 2.63	**0.0005**	28.63 ± 5.08	7.81 ± 1.39	**< 0.0001**
**CA19–9 (U/ml)**	146.26 ± 24.28	47.71 ± 8.61	**0.0001**	107.19 ± 15.80	57.93 ± 12.16	**0.007**	107.15 ± 18.84	28.47 ± 6.08	**< 0.0001**
**Histology differentiation**			**< 0.0001**			**< 0.0001**			**< 0.0001**
well	23(22%)	76(76%)		15(20%)	64(76%)		23(27%)	56(73%)	
poorly	83(78%)	24(24%)		61(80%)	20(24%)		61(73%)	21(27%)	
**overall survival (5 year)**			**< 0.0001**			**0.0003**			**< 0.0001**
alive	46(44%)	76(76%)		34(45%)	60(71%)		39(46%)	60(78%)	
dead	59(56%)	24(24%)		42(55%)	24(29%)		45(54%)	17(22%)	

**Table 3:** Clinical characteristics of human CRC patients according to Beclin 1 expression in NM of the training, testing and independent sets

	training set			testing set			independent set		
	patients with low expression (*n* = 34)	patients with high expression (*n* = 171)	*p* value	patients with low expression (*n* = 21)	patients with high expression (*n* = 139)	*p* value	patients with low expression (*n* = 21)	patients with high expression (*n* = 140)	*p* value
**Age, years**	58.1(31–78)	58.4(34–82)	0.44	53.0(28–70)	58.2(28–84)	**0.03**	61.7(39–92)	61.5(35–87)	0.47
**Sex, male**	15(44%)	91(53%)	0.17	11(52%)	71(51%)	0.46	10(48%)	63(45%)	0.41
**Pathological type**			0.24			0.5			0.24
colon cancer	16(47%)	77(45%)		12(57%)	79(57%)	0.5	16(76%)	93(66%)	
rectal cancer	18(53%)	94(55%)		9(43%)	60(43%)		5(24%)	47(34%)	
**Family history of cancer**			0.41			0.1			0.38
yes	8(24%)	37(22%)		4(19%)	46(33%)		7(33%)	42(30%)	
no	26(75%)	134(78%)		17(81%)	93(67%)		14(67%)	98(70%)	
**T stage**			0.24			**0.02**			**0.03**
T1	0(0%)	5(3%)		0(0%)	11(8%)		0(0%)	11(8%)	
T2	4(12%)	30(18%)		2(10%)	26(18%)		1(5%)	25(18%)	
T3	22(65%)	92(54%)		9(43%)	61(44%)		12(57%)	62(44%)	
T4	8(23%)	44(25%)		10(47%)	41(30%)		8(38%)	42(30%)	
**N stage**			**0.002**			**0.007**			**0.04**
N0	8(23%)	90(53%)		5(24%)	70(50%)		5(24%)	70(50%)	
N1	6(18%)	21(12%)		2(10%)	24(17%)		2(10%)	25(18%)	
N2	11(32%)	39(23%)		5(24%)	22(16%)		8(37%)	20(14%)	
N3	5(15%)	11(6%)		5(24%)	16(12%)		2(10%)	19(14%)	
Nx	4(12%)	10(6%)		4(18%)	7(5%)		4(19%)	6(4%)	
**TNM stage**			**0.0006**			**0.007**			**0.004**
I	3(9%)	27(16%)		1(5%)	22(16%)		0(0%)	23(16%)	
II	5(15%)	63(37%)		4(19%)	48(35%)		5(24%)	47(34%)	
III	15(44%)	60(35%)		10(48%)	50(36%)		10(48%)	51(36%)	
IV	11(32%)	21(12%)		6(28%)	19(13%)		6(28%)	19(14%)	
**CEA (ng/ml)**	39.67 ± 6.23	19.29 ± 4.97	**0.009**	42.66 ± 6.33	20.39 ± 4.20	**0.009**	34.15 ± 4.50	16.35 ± 3.78	**0.01**
**CA19–9 (U/ml)**	140.93 ± 20.11	65.39 ± 12.87	0.001	159.55 ± 20.39	90.72 ± 19.12	0.06	101.48 ± 14.80	57.25 ± 11.79	**0.04**
**Histology differentiation**			0.44			0.06			**0.02**
well	16(47%)	83(49%)		7(33%)	72(52%)		6(29%)	73(52%)	
poorly	18(53%)	88(51%)		14(67%)	67(48%)		15(71%)	67(48%)	
**overall survival (5 year)**			**0.0002**			**0.005**			**0.002**
alive	11(32%)	111(65%)		7(33%)	87(63%)		7(33%)	92(66%)	
dead	23(68%)	60(35%)		14(67%)	52(37%)		14(67%)	48(34%)	

**Table 4:** Clinical characteristics of human CRC patients according to LC3II expression in CT of the training, testing and independent sets

	training set			testing set			independent set		
	patients with low expression (*n* = 124)	patients with high expression (*n* = 81)	*p* value	patients with low expression (*n* = 98)	patients with high expression (*n* = 62)	*p* value	patients with low expression (*n* = 98)	patients with high expression (*n* = 63)	*p* value
**Age, years**	58.9(31–80)	57.4(34–82)	0.34	56.5(28–84)	59.2(37–84)	0.08	60.3(35–92)	63.6(35–87)	0.06
**Sex, male**	59(48%)	47(58%)	0.07	51(52%)	31(50%)	0.4	46(47%)	27(43%)	0.31
**Pathological type**			0.41			0.43			0.32
colon cancer	58(47%)	35(43%)		57(58%)	34(55%)		64(65%)	45(71%)	
rectal cancer	66(53%)	46(57%)		41(42%)	28(45%)		34(35%)	18(29%)	
**Family history of cancer**			0.11			0.32			0.34
yes	31(25%)	14(17%)		32(33%)	18(29%)		31(32%)	18(29%)	
no	93(75%)	67(83%)		66(67%)	44(71%)		67(68%)	45(71%)	
**T stage**			**0.0001**			**< 0.0001**			**< 0.0001**
T1	1(1%)	4(5%)		1(1%)	10(16%)		2(2%)	9(14%)	
T2	13(10%)	21(26%)		11(11%)	17(27%)		10(10%)	16(25%)	
T3	72(58%)	42(52%)		43(44%)	27(44%)		46(47%)	28(45%)	
T4	38(31%)	14(17%)		43(44%)	8(13%)		40(41%)	10(16%)	
**N stage**			**0.001**			**0.0003**			**< 0.0001**
N0	50(40%)	48(60%)		37(38%)	38(61%)		36(37%)	39(62%)	
N1	17(14%)	10(12%)		13(13%)	13(21%)		14(15%)	13(21%)	
N2	32(26%)	18(22%)		20(21%)	7(11%)		18(18%)	10(16%)	
N3	15(12%)	1(1%)		18(18%)	3(5%)		20(20%)	1(1%)	
Nx	10(8%)	4(5%)		10(10%)	1(2%)		10(10%)	0(0%)	
**TNM stage**			**< 0.0001**			**< 0.0001**			**< 0.0001**
I	6(5%)	24(30%)		5(5%)	18(30%)		6(6%)	17(27%)	
II	44(35%)	24(30%)		32(33%)	20(32%)		30(31%)	22(35%)	
III	47(38%)	28(34%)		40(41%)	20(32%)		41(42%)	20(32%)	
IV	27(22%)	5(6%)		21(21%)	4(6%)		21(21%)	4(6%)	
**CEA (ng/ml)**	29.36 ± 5.86	12.43 ± 3.46	**0.005**	32.59 ± 4.81	8.64 ± 3.36	**0.0001**	26.88 ± 4.55	5.91 ± 1.21	**< 0.0001**
**CA19–9 (U/ml)**	137.38 ± 25.39	38.57 ± 10.21	**0.0002**	135.83 ± 22.45	45.62 ± 16.93	**0.001**	100.70 ± 18.66	22.56 ± 4.45	**0.0002**
**Histology differentiation**			**< 0.0001**			**< 0.0001**			**< 0.0001**
well	42(34%)	57(70%)		33(34%)	46(74%)		31(32%)	48(76%)	
poorly	82(66%)	24(30%)		65(66%)	16(26%)		67(68%)	15(24%)	
**overall survival (5 year)**			**< 0.0001**			**< 0.0001**			**< 0.0001**
alive	54(44%)	68(84%)		40(41%)	54(87%)		44(45%)	55(87%)	
dead	70(56%)	13(16%)		58(59%)	8(13%)		54(55%)	8(13%)	

**Table 5:** Clinical characteristics of human CRC patients according to LC3II expression in NM of the training, testing and independent sets

	training set			testing set			independent set		
	patients with low expression (*n* = 33)	patients with high expression (*n* = 172)	*p* value	patients with low expression (*n* = 22)	patients with high expression (*n* = 138)	*p* value	patients with low expression (*n* = 21)	patients with high expression (*n* = 140)	*p* value
**Age, years**	57.4(31–78)	58.5(34–82)	0.29	54.1(28–70)	58.1(28–84)	0.07	64(45–92)	61.2(35–87)	0.16
**Sex, male**	13(39%)	93(54%)	0.06	11(50%)	71(51%)	0.45	11(52%)	62(44%)	0.25
**Pathological type**			0.42			0.35			0.16
colon cancer	14(42%)	79(46%)		11(50%)	80(58%)		16(76%)	93(66%)	
rectal cancer	19(58%)	93(54%)		11(50%)	58(42%)		5(24%)	47(34%)	
**Family history of cancer**			0.37			0.18			0.38
yes	8(24%)	37(22%)		5(23%)	45(33%)		7(33%)	42(30%)	
no	25(76%)	135(78%)		17(77%)	93(67%)		14(67%)	98(70%)	
**T stage**			0.32			0.06			0.1
T1	0(0%)	5(3%)		0(0%)	11(8%)		1(5%)	10(7%)	
T2	4(12%)	30(17%)		4(18%)	24(17%)		1(5%)	25(18%)	
T3	22(67%)	92(53%)		8(36%)	62(45%)		11(52%)	63(45%)	
T4	7(21%)	45(27%)		10(46%)	41(30%)		8(38%)	42(30%)	
**N stage**			**0.008**			**0.01**			**0.04**
N0	8(24%)	90(52%)		6(27%)	69(50%)		5(24%)	70(50%)	
N1	7(21%)	20(12%)		2(9%)	24(17%)		2(10%)	25(18%)	
N2	10(31%)	40(23%)		5(23%)	22(16%)		8(38%)	20(14%)	
N3	4(12%)	12(7%)		5(23%)	16(12%)		2(10%)	19(14%)	
Nx	4(12%)	10(6%)		4(18%)	7(5%)		4(18%)	6(4%)	
**TNM stage**			**0.003**			**0.04**			**0.008**
I	3(9%)	27(16%)		3(14%)	20(14%)		1(5%)	22(16%)	
II	5(15%)	63(37%)		3(14%)	49(36%)		4(19%)	48(34%)	
III	16(49%)	59(34%)		10(45%)	50(36%)		10(48%)	51(36%)	
IV	9(27%)	23(13%)		6(27%)	19(14%)		6(28%)	19(14%)	
**CEA (ng/ml)**	36.00 ± 5.87	20.12 ± 5.11	**0.04**	40.71 ± 6.26	20.53 ± 4.21	**0.01**	34.21 ± 4.49	16.34 ± 3.78	**0.01**
**CA19–9 (U/ml)**	163.74 ± 27.45	90.30 ± 21.42	**0.03**	152.76 ± 28.89	85.25 ± 17.48	**0.03**	101.54 ± 14.79	57.24 ± 11.48	**0.04**
**Histology differentiation**			0.49			0.1			**0.02**
well	16(48%)	83(48%)		8(36%)	71(51%)		6(29%)	73(52%)	
poorly	17(52%)	89(52%)		14(64%)	67(49%)		15(71%)	67(48%)	
**overall survival (5 year)**			**0.0004**			**0.01**			**0.002**
alive	11(33%)	110(64%)		8(36%)	86(62%)		7(33%)	92(66%)	
dead	22(67%)	61(36%)		14(64%)	52(38%)		14(67%)	48(34%)	

**Table 6:** Clinical characteristics of human CRC patients according to Bcl-xL expression in CT of the training, testing and independent sets

	training set			testing set			independent set		
	patients with low expression (*n* = 129)	patients with high expression (*n* = 76)	*p* value	patients with low expression (*n* = 101)	patients with high expression (*n* = 59)	*p* value	patients with low expression (*n* = 103)	patients with high expression (*n* = 58)	*p* value
**Age, years**	57.0(34–78)	59.1(31–82)	0.1	59.5(28–84)	56.4(28–84)	**0.05**	62.8(35–86)	60.9(35–92)	0.16
**Sex, male**	38(50%)	68(53%)	0.35	30(51%)	52(51%)	0.47	25(43%)	48(47%)	0.34
**Pathological type**			0.41			0.32			0.45
colon cancer	31(41%)	62(48%)		36(61%)	55(54%)		40(69%)	69(67%)	
rectal cancer	45(59%)	67(52%)		23(39%)	46(46%)		18(31%)	34(33%)	
**Family history of cancer**			**0.02**			0.31			0.45
yes	23(30%)	22(17%)		17(29%)	33(33%)		18(31%)	31(30%)	
no	53(70%)	107(83%)		42(71%)	68(67%)		40(69%)	72(70%)	
**T stage**			**0.01**			**< 0.0001**			**0.004**
T1	4(5%)	1(1%)		10(17%)	1(1%)		8(14%)	3(3%)	
T2	18(24%)	16(12%)		9(15%)	19(19%)		11(19%)	15(15%)	
T3	36(47%)	78(60%)		32(54%)	38(38%)		25(43%)	49(48%)	
T4	18(24%)	34(27%)		8(14%)	43(42%)		14(24%)	36(34%)	
**N stage**			**< 0.0001**			**0.0001**			**0.0002**
N0	53(70%)	45(35%)		40(68%)	35(34%)		38(66%)	37(36%)	
N1	4(5%)	23(18%)		7(12%)	19(19%)		8(14%)	19(18%)	
N2	14(18%)	36(27%)		8(14%)	19(19%)		11(19%)	17(17%)	
N3	1(1%)	15(12%)		3(5%)	18(18%)		1(1%)	20(19%)	
Nx	4(5%)	10(8%)		1(1%)	10(10%)		0(0%)	10(10%)	
**TNM stage**			**< 0.0001**			**< 0.0001**			**< 0.0001**
I	21(28%)	9(7%)		17(29%)	6(6%)		17(29%)	6(6%)	
II	32(42%)	36(28%)		23(39%)	29(29%)		21(36%)	31(30%)	
III	15(20%)	60(47%)		13(22%)	47(46%)		14(24%)	47(46%)	
IV	8(10%)	24(18%)		6(10%)	19(19%)		6(10%)	19(18%)	
**CEA (ng/ml)**	16.51 ± 5.30	26.30 ± 5.15	0.07	8.54 ± 2.02	31.93 ± 5.39	**0.0002**	6.63 ± 1.23	25.46 ± 4.68	**0.0004**
**CA19–9 (U/ml)**	41.90 ± 7.67	140.79 ± 28.82	**0.0005**	57.83 ± 17.08	126.02 ± 23.27	**0.01**	23.78 ± 4.40	96.22 ± 19.14	**0.0008**
**Histology differentiation**			**< 0.0001**			**< 0.0001**			**< 0.0001**
well	61(80%)	38(29%)		47(80%)	32(32%)		44(76%)	35(34%)	
poorly	15(20%)	91(71%)		12(20%)	69(68%)		14(24%)	68(66%)	
**overall survival (5 year)**			**< 0.0001**			**< 0.0001**			**< 0.0001**
alive	60(79%)	62(48%)		46(78%)	48(48%)		47(81%)	52(50%)	
dead	16(21%)	67(52%)		13(22%)	53(52%)		11(19%)	51(50%)	

**Table 7:** Clinical characteristics of human CRC patients according to Bcl-xL expression in NM of the training, testing and independent sets

	training set			testing set			independent set		
	patients with low expression (*n* = 185)	patients with high expression (*n* = 20)	*p* value	patients with low expression (*n* = 142)	patients with high expression (*n* = 18)	*p* value	patients with low expression (*n* = 147)	patients with high expression (*n* = 14)	*p* value
**Age, years**	58.3(31–82)	58.5(37–75)	0.46	58.4(28–84)	50.7(28–72)	**0.003**	61.5(35–87)	61.6(45–92)	0.5
**Sex, male**	96(52%)	10(50%)	0.44	72(51%)	10(56%)	0.35	65(44%)	8(57%)	0.18
**Pathological type**			0.06			0.07			0.42
colon cancer	81(44%)	12(60%)		83(58%)	8(44%)		99(67%)	10(71%)	
rectal cancer	104(56%)	8(40%)		59(42%)	10(56%)		48(33%)	4(29%)	
**Family history of cancer**			0.18			0.2			0.15
yes	39(21%)	6(30%)		46(32%)	4(22%)		43(29%)	6(43%)	
no	146(79%)	14(70%)		96(68%)	14(78%)		104(71%)	8(57%)	
**T stage**			0.47			**0.02**			0.06
T1	5(3%)	0(0%)		11(8%)	0(0%)		11(7%)	0(0%)	
T2	30(16%)	4(20%)		25(18%)	3(17%)		25(17%)	1(7%)	
T3	103(56%)	11(55%)		65(46%)	5(28%)		67(46%)	7(50%)	
T4	47(25%)	5(25%)		41(28%)	10(55%)		44(30%)	6(43%)	
**N stage**			**0.001**			**0.0007**			**0.03**
N0	95(51%)	3(15%)		72(51%)	3(17%)		73(49%)	2(14%)	
N1	23(13%)	4(20%)		25(18%)	1(5%)		26(18%)	1(7%)	
N2	46(25%)	4(20%)		22(15%)	5(28%)		22(15%)	6(43%)	
N3	11(6%)	5(25%)		16(11%)	5(28%)		20(14%)	1(7%)	
Nx	10(5%)	4(20%)		7(5%)	4(22%)		6(4%)	4(29%)	
**TNM stage**			**0.0003**			**0.006**			**0.002**
I	29(16%)	1(5%)		21(15%)	2(11%)		23(16%)	0(0%)	
II	66(36%)	2(10%)		51(36%)	1(5%)		50(34%)	2(14%)	
III	66(36%)	9(45%)		51(36%)	9(50%)		54(36%)	7(50%)	
IV	24(12%)	8(40%)		19(13%)	6(34%)		20(14%)	5(36%)	
**CEA (ng/ml)**	19.24 ± 4.85	54.47 ± 7.39	**0.0006**	19.70 ± 4.75	51.76 ± 6.39	**0.0007**	16.75 ± 3.72	45.15 ± 4.87	**0.001**
**CA19–9 (U/ml)**	64.08 ± 12.47	209.08 ± 23.62	**< 0.0001**	86.97 ± 18.84	199.73 ± 30.58	**0.0007**	57.80 ± 11.62	128.36 ± 16.55	**0.009**
**Histology differentiation**			**0.004**			0.17			**0.02**
well	95(51%)	4(20%)		72(51%)	7(39%)		76(52%)	3(21%)	
poorly	90(49%)	16(80%)		70(49%)	11(61%)		71(48%)	11(79%)	
**overall survival (5 year)**			**< 0.0001**			**0.0004**			**0.0006**
alive	118(64%)	4(20%)		90(63%)	4(22%)		96(65%)	3(21%)	
dead	67(36%)	16(80%)		52(37%)	14(78%)		51(35%)	11(79%)	

### Autophagic protein signature and OS

**Figure 3:**
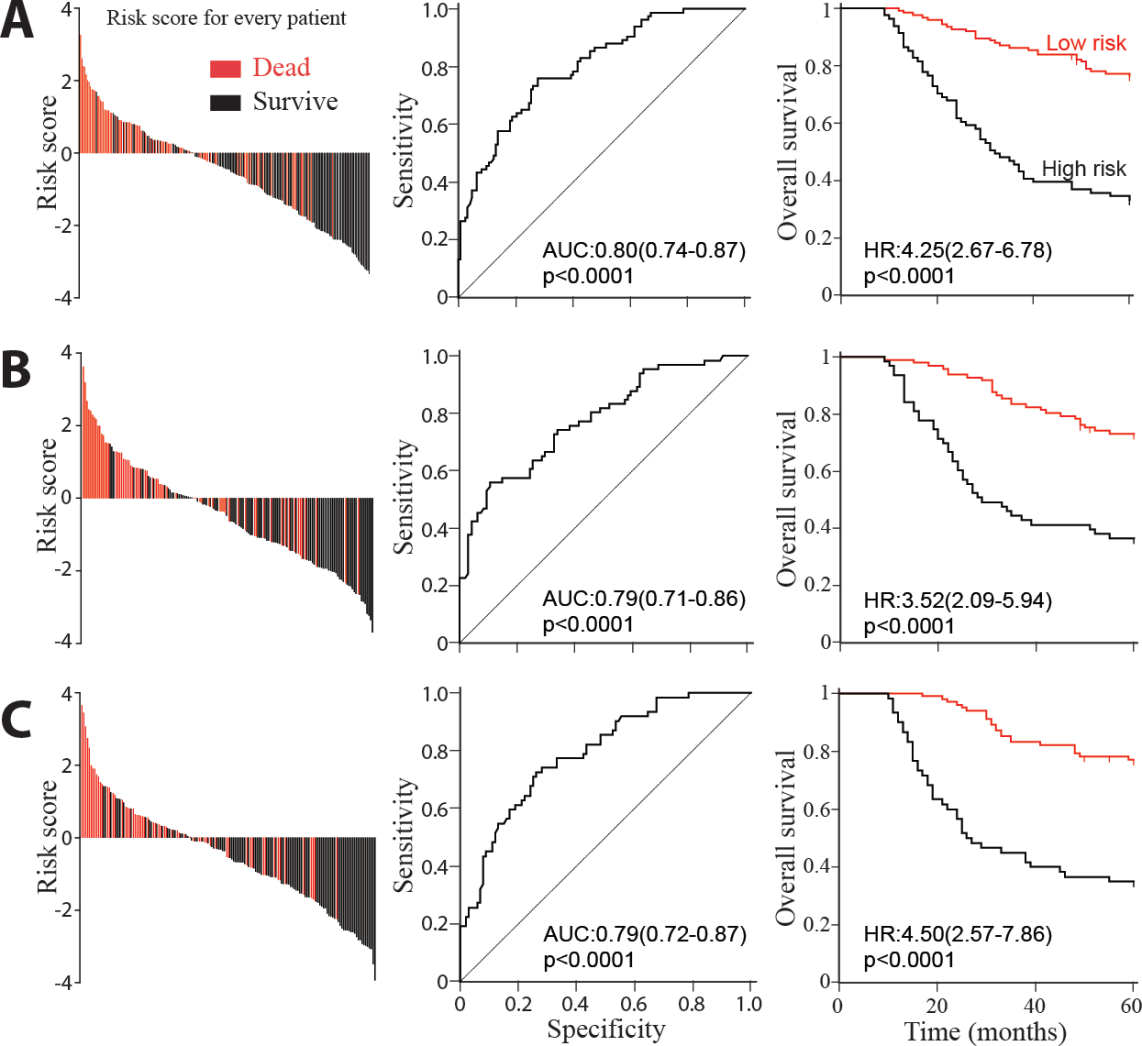
Risk score by the autophagic-protein-based classifier, ROC curves and Kaplan-Meier survival in the training, internal testing and independent validation sets Data are shown as AUC (95% CI) or HR (95% CI). **(A)** Training cohort. **(B)** Internal testing cohort. **(C)** Independent validation cohort.

In order to study the significance of the autophagic proteins for survival prognosis, a formula was developed to measure the risk taking account the strength of all three proteins studied [23]. Risk score = 3.554–(0·248 × Beclin 1_CT_)–(0·451 × LC3B_CT_) + (0.214 × Bcl-xL_CT_) + (0.095 × Beclin 1_NM_) – (0.492× LC3B_NM_) – (0.082 × Bcl-xL_NM_). Using this formula, participants in the training set were categorized into high-risk and low-risk subgroup with risk score = 0 as cutoff value. The details of clinical and pathological characteristics were showed in Table 8. Compared with high-risk patients, low-risk subjects had better OS (Figure 3A, left panel). The prognostic accuracy was assessed by time-dependent ROC analysis (Figure 3A, middle panel). Five-year overall survival was 33% for the high-risk subjects, and 77% for the low-risk patients (HR, 4.25; 95% CI, 2.67–6.78; *p* < 0.0001; Figure 3A, right panel). Same cutoff value were also applied to both internal and external test cohorts. As expected, subjects in both sets with high-risk had worse OS. Five-year OS rate was 73% for the low-risk patients and 37% for the high-risk subjects in internal test cohort (HR, 3.52; 95% CI, 2.09–5.94; *p* < 0.0001; Figure 3B). As for independent validation set, five-year OS rate was 77% for the low-risk subjects and 35% for the high-risk patients (HR, 4.50; 95% CI, 2.57–7.86; *p* < 0.0001; Figure 3C).

**Table 8:** Clinical characteristics of human CRC patients according to high- or low-risk autophagic protein signature in the training, testing and independent sets

	training set			testing set			independent set		
	patients with low risk (*n* = 124)	patients with high risk (*n* = 81)	*p* value	patients with low risk (*n* = 97)	patients with high risk (*n* = 63)	*p* value	patients with low risk (*n* = 101)	patients with high risk (*n* = 60)	*p* value
**Age, years**	58.1(34–82)	58.6(30–81)	0.36	58.3(28–84)	56.3(28–84)	0.14	62.6(35–87)	59.7(39–92)	0.07
**Sex, male**	71(57%)	35(43%)	**0.03**	46(47%)	36(57%)	0.12	44(44%)	29(48%)	0.28
**Pathological type**			0.46			0.36			0.25
colon cancer	56(45%)	37(46%)		58(60%)	33(52%)		71(70%)	38(63%)	
rectal cancer	68(55%)	44(54%)		39(40%)	30(48%)		30(30%)	22(37%)	
**Family history of cancer**			0.23			0.28			0.4
yes	25(20%)	20(25%)		32(33%)	18(29%)		30(30%)	19(32%)	
no	99(80%)	61(75%)		65(67%)	45(71%)		71(70%)	41(68%)	
**T stage**			**0.005**			**< 0.0001**			**< 0.0001**
T1	5 (4%)	0 (0%)		11(11%)	0 (0%)		11(11%)	0 (0%)	
T2	23(19%)	11(14%)		18(19%)	10(16%)		21(21%)	5 (8%)	
T3	71(57%)	43(53%)		49(50%)	21(33%)		48(48%)	26(43%)	
T4	25(20%)	27(33%)		19(20%)	32(51%)		21(20%)	29(49%)	
**N stage**			**< 0.0001**			**0.001**			**< 0.0001**
N0	73(59%)	25(31%)		53(54%)	22(35%)		57(56%)	18(30%)	
N1	15(12%)	12(15%)		18(19%)	8(13%)		19(19%)	8(13%)	
N2	29(23%)	21(26%)		17(18%)	10(16%)		20(20%)	8(13%)	
N3	2 (2%)	14(17%)		6 (6%)	15(24%)		3 (3%)	18(30%)	
Nx	5 (4%)	9(11%)		3 (3%)	8(12%)		2 (2%)	8 (9%)	
**TNM stage**			**< 0.0001**			**0.003**			**< 0.0001**
I	25(20%)	5 (6%)		18(19%)	5 (8%)		21(21%)	2 (3%)	
II	48(39%)	20(25%)		35(36%)	17(27%)		36(36%)	16(27%)	
III	39(31%)	36(44%)		33(34%)	27(43%)		35(34%)	26(43%)	
IV	12(10%)	20(25%)		11(11%)	14(22%)		9 (9%)	16(27%)	
**CEA(ng/ml)**	15.12 ± 3.71	34.24 ± 5.78	**0.002**	13.62 ± 2.75	38.22 ± 5.51	**< 0.0001**	8.84 ± 1.26	35.23 ± 5.60	**< 0.0001**
**CA19–9 (U/ml)**	53.07 ± 9.37	117.41 ± 17.38	**0.0002**	51.82 ± 8.11	127.31 ± 17.78	**< 0.0001**	29.46 ± 4.21	120.58 ± 17.15	**< 0.0001**
**Histology differentiation**			**< 0.0001**			**< 0.0001**			**< 0.0001**
well	84(68%)	15(19%)		67(69%)	12(19%)		71(70%)	8(13%)	
poorly	40(32%)	66(81%)		30(31%)	51(81%)		30(30%)	52(87%)	
**overall survival (5 year)**			**< 0.0001**			**< 0.0001**			**< 0.0001**
alive	95(77%)	27(33%)		71(73%)	23(37%)		78(77%)	21(35%)	
dead	29(23%)	54(67%)		26(27%)	40(63%)		23(23%)	39(65%)	

### Autophagic signature as an independent prognostic factor

**Figure 4:**
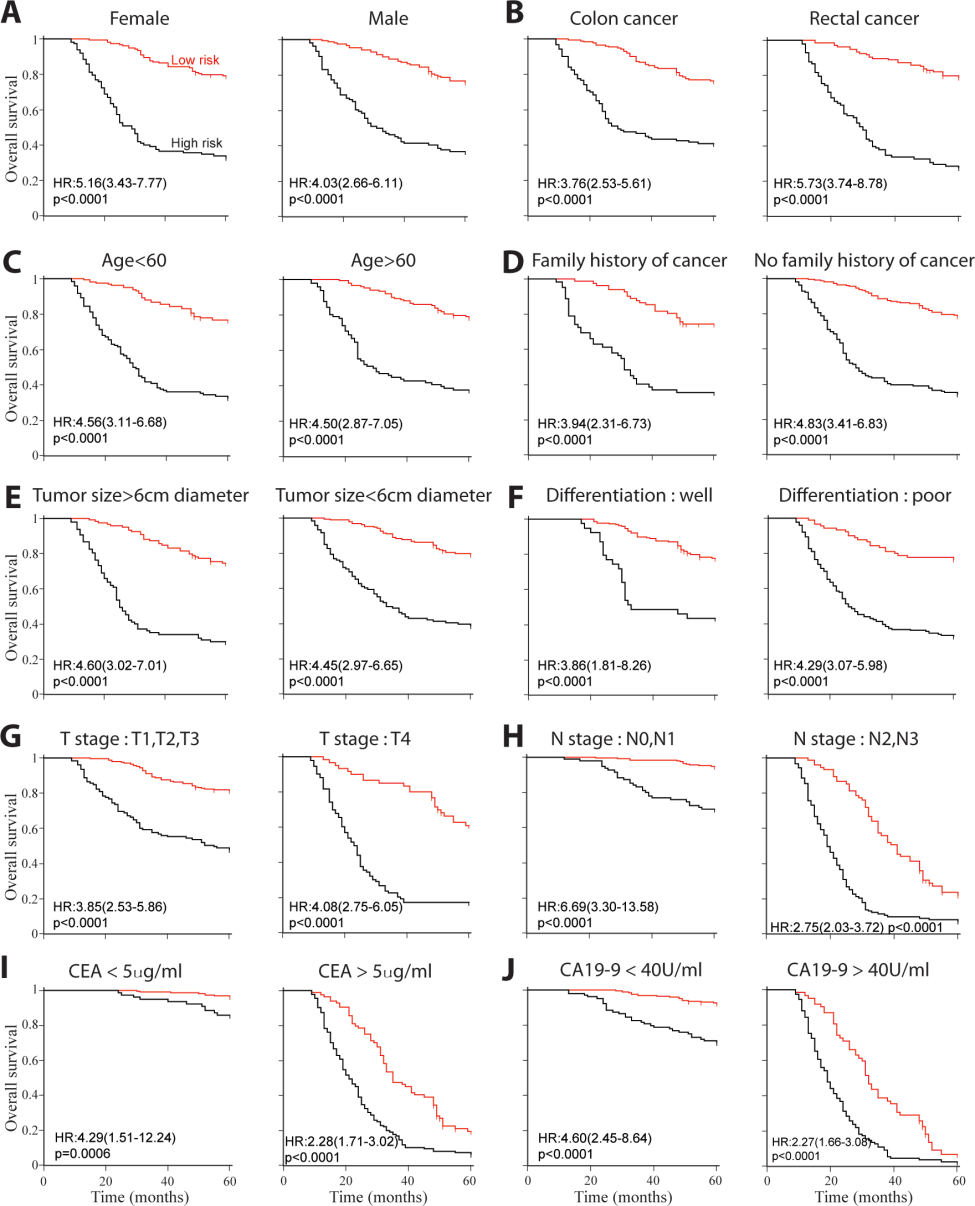
Kaplan-Meier survival analysis for all 526 patients according to autophagic-protein-based classifier stratified by clinical and pathological characteristics **(A)** Gender. **(B)** Cancer location. **(C)** Age. **(D)** Family history of cancer. **(E)** Tumor size. **(F)** Tumor differentiation. **(G)** T stage. **(H)** N stage. **(I)** CEA concentration. **(J)** CA19–9 concentration.

**Figure 5:**
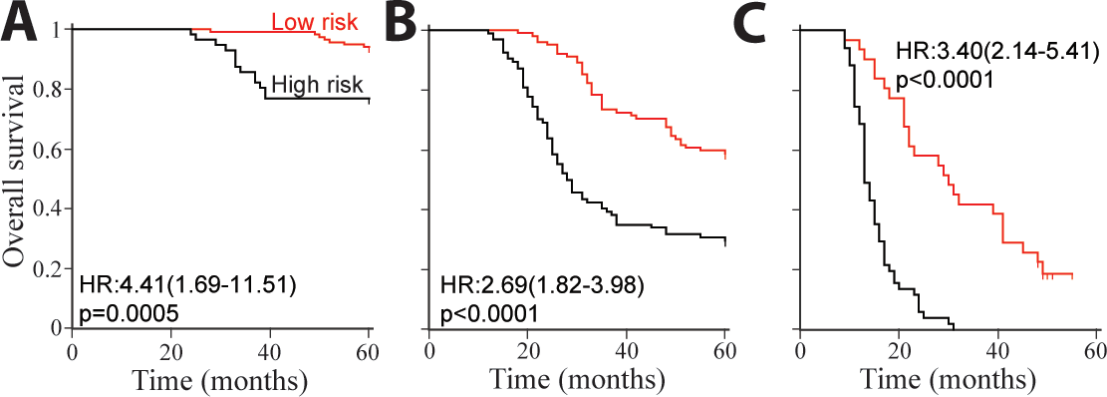
Kaplan-Meier survival analysis for patients with low-risk or high-risk scores in the training and validation sets, which were stratified by tumor stage **(A)** Overall survival of stage II patients, *n* = 172. **(B)** Overall survival of stage III patients, *n* = 196. **(C)** Overall survival of stage IV patients, *n* = 82.

We next conducted multivariable Cox regression analysis to evaluate whether the prognostic ability of this autophagic protein signature was independent of pathological and clinical factors (Table 9). Selected characteristics included gender (Figure 4A), tumor location (Figure 4B), age (Figure 4C), family history of cancer (Figure 4D), tumor size (Figure 4E), tumor differentiation (Figure 4F), T stage (Figure 4G), N stage (Figure 4H), CEA concentration (Figure 4I), CA 19–9 concentration (Figure 4J) and autophagic signature. We also performed stratified analysis in TNM stage II (Figure 5A), III (Figure 5B) and IV (Figure 5C) patients from all enrolled patients to assess the survival prognosis ability of autophagic protein signature within the same clinical stage. Patients in TNM stage I were excluded because the limited participants. Compared with low-risk subjects, high-risk patients had worse OS in all individual stages. These result demonstrated autophagic protein signature was an independent prognostic factor for patients with colorectal carcinoma.

**Table 9:** Multivariable Cox regression of autophagic signature and overall survival in all 526 patients we calculated hazard ratios and *p* values with sex, tumor location, age, family history of cancer, tumor size, tumor differentiation, T stage, N stage, TNM stage, CEA concentration, CA 19–9 concentration and the autophagic signature as covariates. Only variables that were significantly associated with overall survival are presented.

overall survival	Hazard ration (95% Cl)	*p* value
autophagoc signature (high vs low)	1.75(1.46–2.09)	< 0.0001
CA19–9 (high vs low)	1.04(1.03–1.06)	0.001
T stage (T2,T3 vs T0,T1)	1.42(1.08–1.87)	0.02
N stage (N2,N3 vs N0, N1)	1.80(1.42–2.28)	< 0.0001
TNM stage (III, IV vs I, II)	2.13(1.44–3.15)	0.0002
tumor differentiation (well vs poor)	0.56(0.39–0.82)	0.003

### Comparison of autophagic-protein signature and TNM stage

**Figure 6:**
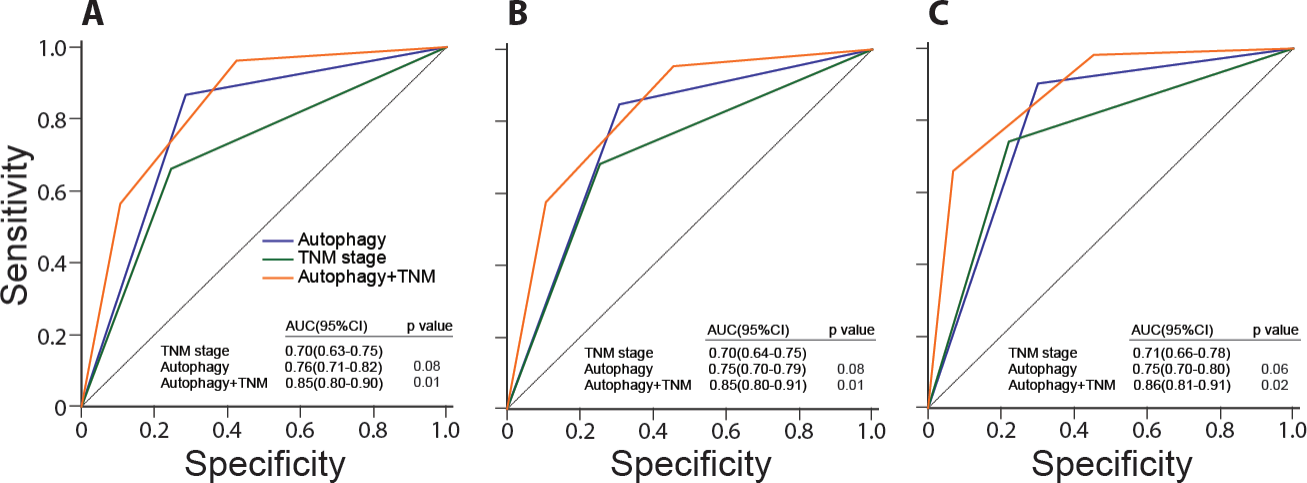
Comparisons of the sensitivity and specificity for the prediction of overall survival by the combined autophagic-protein signature and TNM stage model, the TNM stage alone model, and the autophagic-protein signature alone ROC curves in the training set **(A)**, internal validation set **(B)**, and independent set **(C)** *p* values show AUC of the combined autophagic-protein signature and TNM stage model versus AUC of the TNM stage alone model or the autophagic-protein signature alone model.

In clinical practice, TNM staging system is believe to be the critical prognostic determinant for patients with cancer. ROC analysis suggested that our autophagic protein signature had a similar survival prognostic ability as TNM stage (Figure 6). We then created a prognostic model combining autophagic protein signature and TNM stage based on the internal training set [24, 25]. It had a better prognostic value than either TNM stage or autophagic protein signature alone in the training set (Figure 6A), which were corroborated in the internal validation (Figure 6B) and independent sets (Figure 6C).

## DISCUSSION

In this study, our results demonstrated high frequency of defective mitochondria was correlated with worse OS in CRC. The expressions of Beclin 1, LC3B and Bcl-xL in both tumor area and adjacent noncancerous mucosal region were also associated with overall survivals. Furthermore, we developed an autophagy protein based classifier as a more sensitive prognostic tool. This signature was an independent factor for OS and combined it with TNM stage could improve the prognostic efficacy significantly. Our results suggest that autophagy play an important role in the clinical cancer progression. Moreover, in situ analysis of autophagic related proteins could be valuable in survival prognosis and potential drug targets in the treatment of CRC and possibly other types of cancers.

Mitochondria are highly dynamic organelles through changes in overall mass, interconnectedness, and sub-cellular localization [26], deficiencies in autophagy can cause the accumulation of defective mitochondria, which may subsequently induce DNA damage, oxidative stress and chromatin instability [27, 28]. Most of these results came from basic research, here the frequency of accumulated defective mitochondria in human tissues were quantified for the first time. We observed defective mitochondria in all 205 samples examined; further analysis suggested that the frequency of defective mitochondria was robustly correlated with clinical progress and strongly associated with clinical outcomes in human colorectal carcinoma. These data suggested the metabolic reprogramming of cancer cells were very common phenomena in CRC, the accumulated defective mitochondria might reflect the nutrient uptake activity in cancer cells. With the development of cancer, this reprogrammed metabolic activities could provide extra energy for tumor proliferation. As a result, high frequency of defective mitochondria indicate poor OS in CRC patients.

Although dysregulation of Beclin 1 and LC3 have been investigated in a wide variety of tumors, the role of these proteins in colorectal carcinoma was still controversial, especially when the survival prognosis was involved [29]. It was first demonstrated high Beclin 1 expression was associated with good OS in advanced colon cancer [30], while Guo et al. showed patients with low Beclin 1 expression had longer progression free survivals [31]. As for LC3, it was reported there are three different staining patterns of LC3A with opposite significant in survival prognosis [32]. Although Bcl-xL could regulate the activity of Beclin 1 and control mitochondrial quality [33], no study focus on the role of Bcl-xL in prognosis had been ever reported. Here we detected the expressions of Beclin 1, LC3B and Bcl-xL in both tumor and adjacent noncancerous regions from 526 CRC patients. Interestingly, although the expressions between these three proteins were correlated in tumor region, these correlations were not as robust as those in the noncancerous tissues. These results supported the hypothesis that the dynamic autophagic processes in the center of tumors were different from those in the adjacent noncancerous mucosal tissues. In our study, Beclin 1 and LC3B were more likely under-expressed in the central tumor area compare to those in the adjacent noncancerous mucosal regions; while Bcl-xL showed the reversed expression patterns. In addition, their expressions were strongly associated with survival. Furthermore, although the adjacent noncancerous areas were usually treated as “normal”, the expression patterns of autophagic proteins could also predicted, but in a much less extent, clinical outcome. These results suggested that the autophagic activities in the adjacent noncancerous area was strongly affected by the tumor microenvironment, and might play a role in promoting tumor proliferation.

Several mechanisms were introduced to explain the tumor-suppressing effect of autophagy. First, lack or inhibition of autophagy resulted in up-regulation of ROS [7], this could cause several types of DNA damage such as polyploid nuclei, increased double-strand breaks and gene amplification [34]. The accumulation of DNA damage in cells made them more susceptible during cancer development [35]. Second, it is reported that oncogenic signals were activated during autophagic process and established oncogene induced senescence [36]. Since senescence was characterized as a key obstacle during the development of tumor in many cancers [37], this also could partly explain how autophagy is involved in tumor suppression. Third, many known anti-oncogenes like *Ampk, Lkt, Pten,* and *LKB1* usually functioned as positive effectors in the autophagic process [38–41], whereas many oncogenes, including the class I *PI3K* and *Akt* can suppress autophagy [42]. Therefore, the competition between oncogenes and tumor suppression genes might partly reflected by the autophagic activity. Furthermore, autophagy reduced inflammation and intratumoral necrosis which is essential for cancer development [43]. Accordingly, deficiency or inactivation of autophagic activity could eventually increase the susceptibility of cancer and characterized as a valuable prognosis tool [34].

Prognostic evaluation is vital for the formation of appropriate therapeutic strategy. In clinical practice, TNM staging system is the major decisive factor for prognosis in CRC patients currently. However, its limitations in predicting the survival time of CRC patients are also very clear giving it has been reported that patients within the same stage show a wide varieties in their clinical outcomes. Our finding of autophagic-protein signature indicates that autophagy can be a powerful prognostic tool in clinic treatment of colorectal carcinoma. In addition, this signature shows predictive value in stage II, III, and IV patients in stratified analysis, so this signature can categorize patients within the same TNM stage into high-risk and low-risk subgroup with remarkably distinct survival prospects, suggesting it can significantly improve the accuracy of CRC prognosis. Further ROC analysis reveals that TNM stage and the autophagic protein signature have similar survival prognostic abilities. TNM staging is done primarily based on the anatomical information, while autophagic protein signature shows the molecular characteristics and supplies different clues from TNM staging. In this study, we illustrated that combination of them were more accurate than TNM staging alone in survival prediction, implying the autophagic protein signature was able to consolidate the prognostic value of TNM staging. Lastly, patients within the same TNM staging colorectal carcinoma could be further stratified into distinguishing risk subgroups based on the autophagic protein signature, and accordingly treated with different intensities strategically to improve the clinic outcomes. Such stratification would result in a more personalized treatment for CRC patients.

The present study also has several limitations. First of all, this is a retrospective research; prospective studies involving long-term follow-up of CRC patients are needed to validate our results. Second, this research was conducted on Chinese patients only; the distribution of clinical characteristics might be different in other areas, making it susceptible to the inherent biases of such a study format. It would have provided more information if other kinds of races are included. Third, only three major autophagic related proteins were analyzed in current study since it is difficult to label all the proteins involved in autophagic processes. However, even with three proteins it is still possible to show significant prognostic efficacy that are supported by previous studies and biologically plausible.

In summary, our results suggest the autophagy related organelle and proteins can be applied in survival prognosis, and therefore, the potential therapeutic targets against cancer. We realize that large-scale, prospective studies are needed to prove our data before this autophagic protein signature can be used in clinical practice, but our research guarantees further studies in both basic and clinical fields. Ultimately, our result is bound to be of great value for the study of other types of tumors.

## METHODS

### Clinical specimens

We obtained 526 pathologically proven primary colorectal carcinoma, all of these tumors were adenocarcinomas. For the training and internal testing set, 365 specimens were acquired from Third Affiliated Hospital of Harbin Medical University between Jan, 2004 and Aug, 2007. Patients without tumor sample from initial diagnosis, previous treatment with any anti-cancer therapy and preoperative death were excluded from this study. Both carcinoma part and matched noncancerous mucosal part (1.5–5.0 cm away from the edge of carcinoma) of all samples were immediately obtained after surgery. Of these 365 samples, 205 were processed for both immunohistochemistry (IHC) and transmission electron microscopy observation. The remaining 160 samples were just for IHC. We included another 161 patients, with the same criteria, from Second Affiliated Hospital of Harbin Medical University between Apr, 2002 and May 2008 as independent validation set. Clinical and histopathological variables were characterized according to the UICC-TNM staging system. The observation time in these cohorts were defined as the interval between initial diagnosis and last time of contact (either death or last follow up). Overall survival (OS), calculated as the period from the date of initial diagnosis to death of the same patient, was used for prognostic analyses. The authors state that they have obtained appropriate institutional review board approval from both two participating hospitals and have followed the principles outlined in the Declaration of Helsinki for human or animal experimental investigations. In addition, informed consent has been obtained from all participants involved.

### Transmission electron microscopy

Tissues were first placed in solution containing 2% paraformaldehyde, 3% glutaraldehyde, and 0.1M cacodylate buffer (pH 7.3) for 1 h. After the samples were fixed, they were washed and treated with 0.1% Millipore-filtered cacodylate-buffered tannic acid, post-fixed with 1% buffered osmium tetroxide for 30 min, and stained *en bloc* with 1% Millipore-filtered uranyl acetate. The samples were dehydrated in increasing concentrations of ethanol, infiltrated, and embedded in LX-112 medium followed by polymerization in a 70°C oven for 2d. Ultrathin sections were cut, stained with uranylacetate and conterstained with lead citrate. Tissue sections were studied at 80kV in JEM 1010 transmission electron microscope (JEOL, USA). Since TEM technique itself may cause sampling artifacts frequently, we randomly selected a minimum of ten different spots from each sample nonbiased. Then the number of both normal mitochondria and defective mitochondria were counted for quantification analyses (the percentage of dysfunctional mitochondria) and every single cell had the same probability to be observed in the study. Usually the data were evaluated by two independent researchers (M.Y. and H.Z). If both of them achieved agreement with the results, the value was determined. If the results were different, the third researcher (B.Z.) would involve in the evaluation and they worked out the final score.

### Immunohistochemistry (IHC)

IHC analyses were performed as previously described [44]. All paraffin sections were first evaluated by H&E staining to choose one proper tumor section including both the border of cancer and cross-sectional area. The FFPE tissue sections were cut and de-paraffinized in xylene and then rehydrated with ethanol solutions. The tissues were subsequently placed in EDTA (pH 8.0) and autoclave for 5 min at 121°C. After the antigenicity was retrieved, the sections were submerged in 3% H_2_O_2_ for 15 min to quench the endogenous peroxidase. The sections were then washed with PBS for three times, incubated with the antibodies overnight against Beclin 1 (Abcam, ab97505, dilution 1:250), LC3B (LC3 isoform B, Abcam, ab48394, dilution 1:300), Bcl-xL (Santa Cruz, H-5: sc-8392, dilution 1:200) at 4°C. The tissues were placed in peroxidase-conjugated streptavidin for 30 min, and the final results can be observed with diaminobenzidine. For negative control, PBS replaced the above primary antibody.

The assessments of immune-staining were evaluated according to the guideline previously reported [9], the staining intensity was scored as follows: strong staining (score 3), moderate staining (score 2), faint staining (score1) and no staining at all (score 0). The distribution of stained protein was defined as the percentage accounting for the whole area in the section and scored as follows: 76–100% (score 4), 51–75% (score 3), 26–50% (score 2), 1–25% (score 1) and negative (score 0). The total expression scores were evaluated by combined the evaluations of both staining distribution and staining intensity, and subjected to overall survival analysis. The results of staining were evaluated by two researchers (M.Y and H.Z). If both of them agreed with the results, the score was determined. If the values were different, the third researcher (B.Z.) would involve in the evaluation and they worked together to get a final score.

### Selection of cutoff scores

In order to determine the cutoff values of Beclin 1, LC3B and Bcl-xL for overall survival, the receiver operating characteristic (ROC) analysis was applied in the training set as reported previously [22]. In brief, by maximizing the sum of specificity and sensitivity and minimizing the overall error and the distance of the cutoff value to the top-left corner of ROC curve, the optimum cutoff value was calculated. Here, the clinical outcome were classified into two categories according to survival conditions; i.e. death because of colorectal carcinoma vs. all the other outcomes like survival, censored or death but from other causes.

### Construction of autophagic signature and TNM prognostic classifier

Stratified analysis was conducted to examine whether the association between autophagic protein signature and overall survivals was independent of stage as previous reported [24, 25]. One prognostic score model including only the autophagic protein signature and TNM stage as covariates were constructed. The regression co-efficient of the autophagic signature in proportional hazards models was divided by the co-efficient of TNM staging, the yielded value was subsequently rounded up or down to an integer number to get the score needed. ROC analysis were performed to compare the prognostic power of the combined model with the TNM stage or autophagic signature.

### Statistical analysis

The relation between clinical characteristics and autophagic protein expression were assessed with χ^2^ test, Student's *t* test, or Fisher's exact test depending on the context. The log-rank test and Kaplan-Meier analysis were applied to measure the overall survival, and hazard ratios. We investigated the prognostic or predictive accuracy of each feature and multi-protein-based classifier using receiver operating characteristic analysis. The area under curve (AUC) analysis was used to measure prognostic or predictive accuracy. All the analyses were performed with MedCalc 13.0 and significance was defined as *p* < 0.05.
